# COVID-19 is spatial: Ensuring that mobile Big Data is used for social good

**DOI:** 10.1177/2053951720952088

**Published:** 2020-08-27

**Authors:** Age Poom, Olle Järv, Matthew Zook, Tuuli Toivonen

**Affiliations:** 1 Digital Geography Lab, Department of Geosciences and Geography, University of Helsinki, Helsinki, Finland; 2 Helsinki Institute of Sustainability Science, Institute of Urban and Regional Studies, University of Helsinki, Helsinki, Finland; 3 Mobility Lab, Department of Geography, University of Tartu, Tartu, Estonia; 4 Department of Geography, University of Kentucky, Lexington, KY, USA

**Keywords:** Mobile Big Data, mobility, COVID-19, spatial data infrastructure, social good, mobile phone data, social media data, privacy

## Abstract

The mobility restrictions related to COVID-19 pandemic have resulted in the biggest disruption to individual mobilities in modern times. The crisis is clearly spatial in nature, and examining the geographical aspect is important in understanding the broad implications of the pandemic. The avalanche of mobile Big Data makes it possible to study the spatial effects of the crisis with spatiotemporal detail at the national and global scales. However, the current crisis also highlights serious limitations in the readiness to take the advantage of mobile Big Data for social good, both within and beyond the interests of health sector. We propose two strategical pathways for the future use of mobile Big Data for societal impact assessment, addressing access to both raw mobile Big Data as well as aggregated data products. Both pathways require careful considerations of privacy issues, harmonized and transparent methodologies, and attention to the representativeness, reliability and continuity of data. The goal is to be better prepared to use mobile Big Data in future crises.

This article is a part of special theme on Viral Data. To see a full list of all articles in this special theme, please click here: https://journals.sagepub.com/page/bds/collections/viraldata

## This crisis is spatial

The current COVID-19 pandemic highlights the strong spatial dynamics of crises. The virus outbreak, mitigation measures to contain it and societal impacts all take place across geography. Hot spots, quarantine, closed borders, video-conferencing, and social distancing are all profoundly about distance, separation, and space. In short, the COVID-19 crisis is spatial and therefore our responses must also be spatial.

We already see changes in the mobilities and socio-spatial behavior of individuals and societies. Countries continue to restrict border crossing, banning international travel and implement national and regional containment measures to address local outbreaks. Governments have taken drastic measures to limit the usual daily mobility of people by temporarily closing factories, schools, retail shops, restaurants, and recreational facilities. People are strongly advised or even required to work from home, and all social gatherings and face-to-face social interactions, both professional and leisure, have been and continue to be banned in many places. In short, the response to the COVID-19 pandemic is the biggest disruption to individual mobilities in modern times. Or, as Oliver et al. (2020) argue, the measures to fight the virus have not been as much pharmaceutical, as they have been geographical.

Studying human mobility, individual movements in space and time, has been part of the human geography agenda since Torsten Hägerstrand in the 1960s (Hägerstrand, 1970). What is different now is the scale and scope of available spatial data. Namely, the avalanche of data on individual activity spaces (geographic areas where people conduct their social activities) for entire populations collected by our mobile devices. Mobile Big Data provides a ready means to study the spread of the virus, understand the changes in people’s daily interactions and mobilities, and track the recovery process. In short, population-wide data on individual activity spaces has ready application during the pandemic: understanding the spread of the virus, evaluating adherence to restrictions and analyzing the broader societal impacts of these policies. Our advocacy for the use of these data, however, is tempered both by our experiences in recent months with the limitations of using mobile Big Data and our unease with the power of these same data to track, surveil and discipline social behavior at the scale of entire populations. The question we pose here is: How can we use mobile Big Data for social good, while also protecting society from social harm? By social good, we mean improvements in the quality of life for the general population rather than individuals or sub-segments. To do so we outline lessons learned from Estonia and Finland, as well as the practices of corporations more globally.

## Pre-COVID-19 mobile Big Data research

Mobile Big Data refers to all Big Data with spatial (geographic location of the event) and temporal (time specification of the event) information. These data reveal behavior of people in space and time via the proxy of unique technological entities, e.g. smart or mobile devices such as mobile phones, public transit cards or sport watches as well as applications used on these devices such as Twitter, Facebook or Google. The ever-growing share of people carrying digital devices provide data that allows tracking of the spatial flows of dynamic populations (Shoval, 2007), but also the activity spaces of individuals across a range of spatial and temporal scales (Järv et al., 2018). These data include call detail records collected by mobile network operators as well data from mobile operating systems (e.g., Android or iOS) that collect significantly denser spatial and temporal data (via GPS and other signals) and is only available to the developers of these systems (e.g., Google or Apple). Geographically located posts on social media platforms such as Twitter and Instagram are a third example of mobile Big Data about people’s activities and attention through their content creation and curation practices (Poorthuis et al., 2019) across space (Toivonen et al., 2019). Finally, there are thousands of mobile applications with location-based features, e.g. weather forecast providers such as The Weather Channel, sports apps such as theScore, ride-sharing platforms and food delivery companies that collect data on the location of individuals more sporadically or surreptitiously. The rollout of 5G networks and the internet of things open further opportunities for mobile Big Data production and collection including the means and opportunity to monitor a population’s location continuously.

Analysis with these data has provided insights on a wide variety of social phenomena and socio-spatial processes, including crisis situations. Examples include, e.g., analysis on population mobility and commuting (Ahas et al., 2015; Järv et al., 2012), detecting functional economic regions (Novak et al., 2013; OECD, 2020), the provision and accessibility to state services (Järv et al., 2018), identifying migration flows (Kamenjuk et al., 2017) and cross-border mobility (Silm et al., 2020a), analyzing (in)equity between population groups and spatial segregation (Mooses et al., 2016; Shelton et al., 2015; Silm et al., 2018), supporting transport solutions (Positium, 2019) and environmental management (Heikinheimo et al., 2020; Poom et al., 2017), characterizing tourist behavior (Campagna et al., 2015; Raun et al., 2016; Saluveer et al., 2020), or reflecting the lived experiences of people in case of disruptions (Shelton et al., 2014). Much of this research is conducted in countries where access to mobile Big Data has been relatively easy. For example, in Estonia (Silm et al., 2020b), the opportunities afforded by mobile Big Data were already recognized in the mid-2000s and applied to public planning, administration (Ahas and Mark, 2005) and tourism monitoring (Ahas et al., 2007).

Mobile Big Data have also been used in health research to study how virus transmission is mediated by human mobility as well as the impact of accessibility on healthcare. For example, Wesolowski et al. (2012) tied the interregional spread of malaria to human travel in Kenya, and Finger et al. (2015) showed how mass gatherings became hotspots for cholera outbreaks in Senegal. Bengtsson et al. (2015) used mobile phone data to improve predictions on the spatial evolution of Haiti cholera epidemic, and Wesolowski et al. (2015) applied similar mobile phone data to map the uptake of preventive healthcare in Kenya. Kraemer et al. (2018) showed that virus transmission models that incorporated social media data resulted in similar epidemiological inferences as traditional models. In short, mobile Big Data can help us better understand the spatial dimensions of social and health phenomena.

## COVID-19 highlights the challenges of mobile Big Data

Given this history of research with mobile Big Data, it is not surprising that a number of projects have worked to apply this knowledge to the COVID-19 pandemic. These include how the virus spreads (Chang et al., 2020), the efficiency of mobility restrictions (Kraemer et al., 2020), and the social acceptance of restriction measures (Statistics Estonia, 2020). However, the current crisis also highlights serious limitations in our readiness to use mobile Big Data and do so responsibly (Benton et al., 2017; Zook et al., 2017).

For example, despite the long research tradition *in Estonia*, mobile Big Data was not accessible to researchers during the COVID-19 pandemic because of ongoing discussions between mobile network operators and data protection agency. These deliberations focused on differing interpretations of Estonia’s Electronic Communications Act and the lack of clarity on a new EU ePrivacy Regulation. This meant that the previous well-functioning collaboration between network operators and researchers was no longer operating, rendering raw data inaccessible. Instead, the Estonian state, in collaboration with mobile network operators, developed an ad hoc solution to monitor the population’s daily mobility, albeit at a relatively high level of aggregation, to track how well people followed instructions to avoid unnecessary mobility (Statistics Estonia, 2020). This was done via quickly developed methodological guidelines designed by the Estonian state and a data intelligence company Positium, applied by the network operators with undocumented methodological details. As a result there was no space for different data aggregation (needed for more sophisticated analysis) or for longer term follow-up of the situation. In sum, the Estonian case highlights how the lack of legal clarity around using mobile Big Data (held by private companies) can result in less useful applications than otherwise might be the case.

On the other hand, *in Finland*, access to mobile phone data has been rather limited all the time due to strict interpretation of privacy related legislation. While mobile network operators have collaborated with researchers and statistics officials, the scale was exploratory rather than operational. Recently, however, a main mobile network operator, Telia, developed an aggregated and anonymized data product allowing mobility analysis at the scale of the entire population. When the COVID-19 pandemic started, the existence of this ready-made data product allowed governmental officials and researchers quick data to uncover changing mobility flows brought about by closing the borders of the capital region and instructing citizens to avoid visiting secondary homes (Järv et al., 2020a, 2020b; Kotavaara et al., 2020). However, the relatively simple data product did not leverage or allow access to individual-level raw data necessary to create custom spatial and categorical aggregations. Moreover, because Telia’s preconstructed data products were designed to answer specific questions, they could not always address the new questions resulting from COVID-19. Further complicating the application of these data products was that the methodology behind them was not transparent enough to understand fully how the resulting values are derived. Thus, even when access to mobile Big Data is available, it may not be structured in ways that fit the specific needs that arise during a crisis.

In addition to lessons from working with national mobile network operators, it is also useful to understand how some *global* companies deployed their mobile Big Data capabilities. Large platform companies such as Apple, Google or Facebook produced ad-hoc data products and visualizations of mobility during COVID-19. This involved local reports based on the aggregated data of customers, including the use of travel modes or visits to various types of places (Apple, 2020; Google, 2020), or population maps for disease forecasting and prevention (Facebook, 2020). However, because methodologies behind these ad hoc data products were “black boxed” (Pasquale, 2015), it is difficult to evaluate their usefulness or potential for further use. Basic questions such as which population groups were represented remained unknown. As Google (2020) noted their reports “…shouldn’t be used for medical diagnostic, prognostic, or treatment purposes. It also isn’t intended to be used for guidance on personal travel plans.” In a very real sense, application of mobile Big Data from these platforms was limited to insiders rather than officials or citizens seeking to identify hot spots or conduct contact tracing. This echoes the experiences in Estonia and Finland, and aptly illustrates boyd and Crawford’s (2012) observation of how Big Data creates “new digital divides”. This also results in very different analyses (profit vs. social good) and creates methodological disharmony (“black box” vs. open science) in processing and publishing results. In short, the semi-opaque methodologies of mobility data products from private companies frustrate efforts in using these data to create applications targeted at the public good. Moreover, lack of transparency about methods exacerbates privacy and surveillance concerns, a particularly important point given platform- or government-led actions for population control during the containment phase of the COVID-19 crisis (see Kitchin, 2020).

## Improving mobile Big Data systems to promote social good

These examples of mobile Big Data use during the COVID-19 pandemic demonstrate the need to re-evaluate the public–private relationship with mobile Big Data, particularly those associated with individual level mobilities. If we have to accept the production, collection, and monetizing of personal digital footprints in the Age of Surveillance Capitalism^1^ (Zuboff, 2019), how might also we increase the social good of these data? As outlined here, current practices are occasional, ad hoc and opaque, organizationally fragile, and lack an overall strategic approach to key questions around privacy and surveillance. Towards this goal, we propose two strategic pathways to apply mobile Big Data for social good. Just as the COVID-19 pandemic is spatial, so too are many other important social phenomena including gentrification, segregation and accessibility, and understanding differences in mobility can provide welcome insight.

First, we call for *transparent and sound mobile Big Data products* that provide relevant up-to-date longitudinal data on the mobility patterns of dynamic populations. To help increase their usefulness, data products should be transparent about their production methodology, and ensure easy access and stability. While much of the data in statistical offices are transparent, accessible and stable, they are less useful for studying the mobility and activity spaces of people especially in fast-changing phenomenon like the COVID-19 pandemic. Instead, the dynamics of mobility are more easily studied via mobile Big Data that are mostly collected and processed by private companies. Not surprisingly, in the recent months, there have been several calls for privately owned large-scale mobile Big Data to be shared for public health purposes (Buckee, 2020; Ienca and Vayena, 2020; Oliver et al., 2020). While we agree with these calls, we would extend them and argue that availability of these types of data should extend beyond the needs of health sector and this particular pandemic of COVID-19. Of course, given the sensitivity of mobile Big Data, respecting personal privacy is paramount. Possible approaches might include in-house aggregation by the data providers and testing for deanonymization before sharing data products with strict accessibility rules. Products should be developed, tested, and used during normal times (i.e. non-crisis situation), to provide a base for their quick application when needed. To facilitate international comparisons and analysis, data products should use coordinated methodologies and joint data access platforms such as offered by Eurostat, the EU-level statistical office.

Second, building from the idea of ready-made, aggregated data products, we also see *the need to develop trustworthy platforms for collaborative use of raw individual level data*. Secured and privacy-respectful access to near real-time raw data is needed for developing and testing sound methodologies for the above-mentioned data products. This would help bridge the Big Data digital divide, enable scientific innovation, and offering needed flexibility in responding to unanticipated questions on changing locations and mobilities in case of crises. Bottom-up initiatives of data donations and individual data control like MyData^2^ are useful, but do not yet solve the problem. These initiatives tend to involve people with higher knowledge, energy, and capacity to manage their personal data and generally miss more marginalized groups resulting in biased conclusions about society. Models for allowing vetted researchers to work with anonymized individual level data at firewalled data centers include the US Census Bureau’s Research Data Center or the research services of Statistics Finland/Statistics Estonia. Incentivizing or compelling corporations to contribute data would be challenging but might be achieved via social responsibility programs, or legislation requiring data contributions to be allowed to operate within a legal jurisdiction. To be clear, we do not view this as simple to achieve, particularly as we weigh what kind of institution might best fill this role. National Libraries? Academies of Science? United Nations? An independent non-profit with representation from stakeholder communities (users, governments, business, etc.) akin to ICANN^3^? And this is but the first of many questions. How might any of these institutions avoid capture by powerful players? Equally, how is “social good” defined and operationalized in practice when granting access to researchers or state actors? While these questions remain to be answered, we argue that addressing them via public debates and academic discourses will leave us better prepared for the next crisis even if progress on these two pathways falls short.

## Four axioms for moving forward

Summing up, there are important lessons to take from the current pandemic and about the challenges of accessing and making useful applications of mobile Big Data. While we have sketched out two pathways forward, we recognize that these are not the only options available. Therefore we will end this commentary by sketching out four axioms we believe to be fundamental in creating a common framework for gathering and using mobile Big Data.

First, *we need harmonized and representative data about human mobility for better crisis preparedness* and social good in general. While an ad-hoc analysis strategy in Estonia and Finland has been rather satisfactory in case of the COVID-19 pandemic, it also suffered from a limited ability to address specific actions and questions. Second, *methodological transparency about mobile Big Data products (particularly coming from private companies) are vital for open societies and for capacity building*. The present trend in which “corporate secrecy expands as the privacy of human beings contracts” (Pasquale, 2015: 26) must be countered so that mobile data is used for social good rather than simply corporate profit. Third, *access to mobile Big Data to develop feasible methodologies and baseline knowledge for public decision-making is needed before the next crisis occurs*. As our examples outline, solving data access issues can provide new opportunities for increasing the expertise and capacity of researchers working on human mobility and other socio-spatial phenomena. It is vital that the related developments and discussions happen in “normal” times rather the high-pressure and compressed timelines of a crisis. Fourth, and the most relevant of all is *recognizing the fundamental spatiality of the current COVID-19 crisis and crises more generally*. The COVID-19 pandemic (and every other social phenomenon) has deep and important spatial dimensions that spatial data can help us better understand and address in ways that promote social good. The challenge, of course, is doing so responsibly (Zook et al., 2017) via sound and transparent methods and collaborations across trustworthy platforms that do not normalize a lack of spatial privacy.

## Notes

1 While not wishing to reify this as inevitable—after all systems of data collection, surveillance and monetization are social constructions—we do take the pragmatic approach that these kinds of practices will be hard to eliminate in the short term.

2 An open platform for voluntary personal data provision and management that enables commercial use of the data while preserving privacy (https://mydatafi.wordpress.com/).

3 Internet Corporation for Assigned Names and Numbers.
